# A Case of Extrapulmonary Tuberculosis Two-Ways

**Published:** 2019-02-26

**Authors:** Joseph Cherabie, Nicolas Ojile, Thomas A. Moore

**Affiliations:** 1 University of Kansas School of Medicine-Wichita, Department of Internal Medicine; 2 Infectious Disease Consultants, Wichita, KS

**Keywords:** tuberculous, Mycobacterium tuberculosis, encephalitis

## INTRODUCTION

Tuberculosis (TB) is a widespread epidemic. The World Health Organization estimated 10.4 million cases of tuberculosis in 2016, with 490,000 new cases of multi-drug resistant (MDR) TB.^1^ The primary presentation of TB is pulmonary. However, in the United States in 2014, 21% of TB cases were extrapulmonary with the most common sites in descending order of incidence being TB lymphadenitis (38.2%), pleural (16.3%), bone and/or joint (10.4%), peritoneal (5.7%), genitourinary (5%), meningeal (4.5%), and laryngeal (0.2%).^2^

## CASE REPORT

A 72-year-old Laotian male presented from an outside facility with altered mental status and fever. His medical history included hypertension, gout, and stage 3 chronic kidney disease. The fever was cyclic in nature for one-month duration, reaching a peak of 103^o^ F and being consistently above 100° F three days prior to presentation. The patient’s altered mental status developed over those three days, which consisted of confusion and visual hallucinations. During this time, the patient developed abdominal distension with ascites, which resolved on its own before a paracentesis could be performed. The patient’s family reported an unintentional weight loss of 15 pounds over a six-month period.

**Figure 1:**
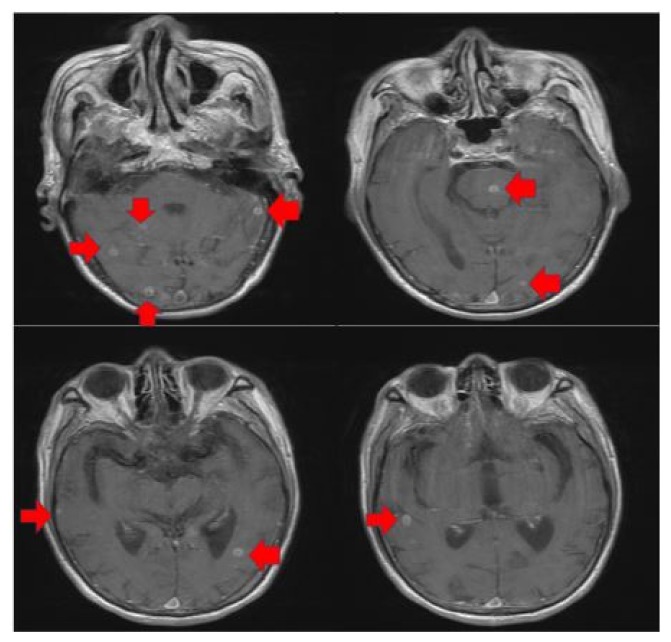
Magnetic resonance imaging showed multiple small intra-parenchymal lesions with bilateral cerebral hemispheric involvement and a small brain stem lesion.

Prior workup outside of our facility included computerized tomography (CT) of the head, chest, abdomen, and pelvis. The patient’s abdomen/pelvis showed omental caking and ascites, suggestive of peritoneal carcinomatosis. CT of the head and magnetic resonance imaging (Figure 1) showed multiple small intra-parenchymal lesions with bilateral cerebral hemispheric involvement and a small brain stem lesion, believed to be either metastases or abscesses.

Upon arrival to our facility, the patient was acutely encephalopathic with persistent visual hallucinations. There was no report of any abdominal pain, nausea, vomiting, or diarrhea. On physical exam, he was febrile and had a soft, non-distended abdomen with no hepato-splenomegaly, fluid wave, shifting dullness, or any signs of residual ascites. He was oriented to person but not to place or time and showed no focal motor or sensory deficits. The patient met sepsis criteria with altered mental status, hypotension, fever, and leukocytosis. Two sets of blood cultures were collected and the patient was started empirically on dexamethasone, vancomycin, and ceftriaxone for suspected central nervous system infection and encephalitis. Lumbar puncture was not performed in light of the intra-parenchymal brain lesions and presumed increased intracranial pressure. Interventional radiology was consulted to assess if any ascitic fluid or omental biopsy could be obtained for analysis, however, neither could be found nor deemed safe for biopsy. Hematology-oncology ordered carcinoembryonic antigen, prostate-specific antigen, and a CA19.9 radioimmunoassay.

On day two of admission, the patient remained febrile and testing was expanded to include a TB QuantiFERON^®^ Gold test, toxoplasma IgG and IgM, histoplasma urine antigen, and human immunodeficiency virus (HIV) 1 and 2 antibodies. The patient continued to be encephalopathic with no change in his physical exam.

By hospital day three, mental status began to improve with the patient being alert, awake, and oriented to person, place, and time. The patient’s TB QuantiFERON^®^ Gold test was positive, with the patient’s family denying any history of or exposure to TB. HIV antibodies were negative. Chest x-ray was performed in light of the positive Quantiferon test and showed no signs of active TB or cavitary lesions. Urine histoplasma antigen was negative, as well as the full oncology workup. Blood cultures at that time showed no growth. Toxoplasma IgG was positive, while his IgM was negative. Infectious diseases assessed the need to treat for TB in light of a positive Quantiferon test but a negative chest x-ray, and whether to treat toxoplasma with brain lesions but a negative IgM.

On day six, the patient underwent brain biopsy procedure to assess whether the lesions represented underlying malignancy or infection. The initial pathology results showed caseating granuloma within the brain lesions, most likely representing TB. Cultures of the lesions were taken with mycobacterial cultures. The patient underwent a repeat TB QuantiFERON^®^ Gold test which came back negative, but in spite of this, the patient was started on quadruple therapy for TB empirically due to the pathology from his brain biopsy.

One month after the biopsy, Mycobacterium cultures returned positive for Mycobacterium tuberculosis, confirming the diagnosis of Mycobacterium tuberculosis encephalitis.

## DISCUSSION

This case exhibited two different presentations of extrapulmonary TB: presumed abdominal/peritoneal TB followed by TB meningitis/encephalitis. The most common pathogenesis of both abdominal/peritoneal TB and TB meningitis/encephalitis is from hematogenous spread from primary pulmonary TB, each being a form of disseminated TB; however, only 15 – 25% of abdominal TB patients and 40% of TB meningitis patients have concomitant pulmonary TB.^3^ Extrapulmonary TB most often occurs months after pulmonary TB.

Risk factors for extrapulmonary TB in the general population include HIV infection, young age, old age, especially if age is greater than or equal to 65, Asian or African origin, and female sex.^4^ Risk factors for abdominal/peritoneal TB specifically include alcoholic liver disease and cirrhosis, continuous ambulatory peritoneal dialysis for chronic renal failure, diabetes mellitus, and Bacillus Calmette-Guérin therapy for superficial bladder carcinoma.^5^,^6^ Our patient had two risk factors for TB meningitis, age greater than 65 and coming from southeastern Asia, but denied any of the abdominal TB specific risk factors.

The clinical presentation of abdominal TB is very non-specific, with the most common presenting symptoms being fever, weight loss, abdominal pain, diarrhea, and abdominal distension.^7^ Peritoneal TB presents in one of three forms: wet type with ascites, fibrotic type with localized abdominal swelling, or dry-plastic type with the typical “doughy” abdomen, often with omental caking. Typically, patients presenting with peritoneal TB move through the three types in successive fashion, starting with wet type then moving onto fibrotic type to dry plastic types, and this succession occurs in a subacute fashion over a time period of weeks. Definitive diagnosis of peritoneal TB comes from fluid cultures or biopsy from the abdomen.^5^,^8^ Our patient’s initial presentation was typical of wet type with ascites which resolved on its own and progressed to dry plastic type with omental caking.

TB meningitis is a more common presentation of extrapulmonary TB with the typical clinical presentation being similar to that of other forms of meningitis: stiff neck, headache, fever, vomiting, altered consciousness occurring in greater than 50% of patients, and anorexia, personality changes, weight loss, night sweats, coma, and plegia or paresis being less common.^9^ Our patient initially presented with headache, fever, altered level of consciousness, anorexia, personality changes, and weight loss. Definitive diagnosis of TB meningitis typically is achieved with detection of Mycobacterium tuberculosis in the cerebrospinal fluid via lumbar puncture, either via smear microscopy, mycobacterial culture, or nucleic acid amplification test. Our patient instead underwent brain biopsy with caseating granuloma being found on biopsy.

Of note, our patient’s initial TB QuantiFERON^®^ Gold was reactive. However, when the test was repeated, it was non-reactive. The ultimate diagnosis came from culture from the patient’s brain lesions which grew on mycobacterial culture 30 days after the initial biopsy. TB QuantiFERON^®^ Gold has shown a specificity ranging from 96 – 98.1%, and a sensitivity ranging from 67 – 89%.^10^ The U.S. Centers for Disease Control and Prevention caution against using a negative QuantiFERON^®^ Gold result as a method of ruling out TB in particular populations, especially if there is a high clinical suspicion of TB as there was in this case.^2^

## CONCLUSION

This case highlighted the need for a high level of clinical suspicion with regards to diagnosing TB in a patient who is at high risk for developing extrapulmonary TB due to coming from an endemic region for TB. Our patient had quite the typical presentation for abdominal TB that had progressed from wet to dry type. Abdominal ascites and intra-parenchymal brain lesions cast a wide differential, including more common liver pathologies and malignancy. Still, TB should be included in that differential and investigated when risk factors are present, as they were in this case.
